# The Relationship Between Work Engagement and Job Performance: Psychological Capital as a Moderating Factor

**DOI:** 10.3389/fpsyg.2022.729131

**Published:** 2022-02-17

**Authors:** Jin Yao, Xiangbin Qiu, Liping Yang, Xiaoxia Han, Yiying Li

**Affiliations:** ^1^School of Education Science, Huaiyin Normal University, Huai’an, China; ^2^School of Education, Huzhou University, Huzhou, China; ^3^School of Psychology, Nanjing Normal University, Nanjing, China

**Keywords:** work engagement, job performance, psychological capital, moderating, U-shaped relationship

## Abstract

Based on the job demands-resources model, this study explored the relationships of work engagement, job performance and psychological capital in industry employees. A total of 399 IT programmers were recruited and completed the work engagement scale, knowledge employee job performance scale and psychological capital questionnaire. The results showed that: (1) There is a relationship between work engagement and job performance, which may not be linear but inverted U-shaped, and (2) psychological capital plays a moderating role in the inverted U-shaped relationship between work engagement and job performance.

## Introduction

Traditionally, enterprises have developed internally and externally to maintain a competitive advantage in industry. By purchasing high technology and other resources, the entry threshold to industry could be improved while the cost of similar enterprises could be increased and their competitiveness reduced. The continuous development of human resources could also improve work efficiency and the potential for innovation. However, in the current era of “Internet Plus” which is the integration of the internet and traditional industries through online platforms and information technology (IT). Therefore, external resources and technology can reach a common state through the Internet. As such, the mechanisms for improving the threshold of entry do not now bring more competitive advantages to enterprises. In contrast, the development of internal human resources has become an important source of competitive advantage and innovation for enterprises today (Yao and Yang, 2016). In the past, internal human resource development measures mainly included: (1) actively carrying out knowledge and skills training and improving the professional quality of employees to improve work efficiency and innovation ability, and (2) increasing the engagement of employees in work and increasing the total amount of completed business to maintain the performance of the whole enterprise (Tang and Sun, 2011).

Although these measures previously achieved some positive results, they may not be able to do so in these “Internet Plus” times and may even be a hindrance. The main reason for this is that the knowledge and technology represented by information technology have increased greatly. At the same time, the speed of updating is also very fast, and the cost of knowledge and skills training has been improved. In addition, increasing the work engagement of employees also increases the pressure placed on them, which is likely to lead to them falling into cycles of excessive fatigue and burnout (Rycroft and Kash, 2016). In the new era, performance improvement brought by the training of knowledge and skills and the increase of work engagement has been found to be lacking, which makes researchers and practitioners doubt that the cost of increasing investment can achieve the expected benefits. However, at present, the emphasis of enterprises on increasing the work Engagement of employees to achieve the growth of human capital remains unchanged (Tang and Sun, 2011), in order to solve this contradiction, it is necessary to find a new sustainable development of internal resources to promote the improvement of job performance. In the process of finding such resources, many researchers have paid attention to the role of psychological capital. Psychological capital refers to an individual’s positive state of psychological development, which is manifested as: (1) when facing challenging work, having confidence (self-efficacy/self-confidence) and making the necessary efforts to achieve success, (2) having a positive attribution (optimism) to present and future successes, (3) persevering in goals and adjusting the approach (hope) to achieve goals and successes, and (4) when faced with adversity and problems, persevering, recovering quickly and surpassing difficulties (resilience) to achieve success (Luthans et al., 2008).

Psychological capital can maintain employee working motivation and alleviate job burnout. However, employees with higher psychological capital will actively connect with other resources, learn new skills related to work, and promote individual growth, development, and performance improvement (Wu et al., 2012). Psychological capital has a strong role in promoting job performance. In this context, what role can psychological capital play in the contradiction between input and output? Therefore, under the background of “Internet plus,” this paper explores the relationship between employee’s job involvement and job performance and the effect of psychological capital, which can provide a way to solve the contradiction between employee’s input and output.

The theoretical basis of this study are job demands-resources model. According to the job demands-resources model, when the job requirements and work resources match, the employee’s work efficiency is higher, on the contrary, the work efficiency is low (Liu et al., 2020). To some extent, the two theories reveal the relationship between work engagement and job performance, and the role of psychological capital in it, the details are as follows:

### The Relationship Between Work Engagement and Job Performance

Work engagement is a positive and complete emotional and cognitive state related to work, associated with the characteristics of persistence and dispersion (Li and Ling, 2007; Aldabbas et al., 2021). Based on findings from previous studies, there remains debate regarding the relationship between job involvement and job performance. Some researchers have proposed that with an increase of work engagement, employee emotional, cognitive and forward-looking behaviors will positively improve, which will also lead to an increase in job performance (Wang and Chen, 2020).

However, some other researchers argue that an increase in work engagement does not necessarily lead to the continuous growth of job performance, which may reflect an inverted U-shaped relationship (Bouckenooghe et al., 2021). For example, the job demands-resources model (JD-R) proposed by Demerouti et al. (2001) proposes that the factors that affect the job performance of employees are due to two aspects: work requirements and work resources. Work requirements refer to the physical, psychological, social and organizational requirements of employees, which draw on their continuous physical and/or psychological (cognitive and emotional) efforts and/or skills including their ability to deal with work pressure, work engagement, emotional exhaustion, work-life conflict and so on. Work resources refer to the physical, psychological, social and organizational resources that can be used by employees to achieve work objectives, including the resources owned by individuals themselves, as well as the social and organizational resources that can be obtained. These include workers’ cognitive styles, self-confidence and behavior models, leadership, support from colleagues, family and friends, promotion opportunities, salary, working atmosphere, diversity of tasks, and so on (Demerouti et al., 2001; Qi and Wu, 2018).

When work requirements match an individual’s work resources, increasing work engagement will improve job performance. However, if the work requirements exceed an individual’s work resources and increase work engagement, this will fail to bring about an improvement in job performance and will also result in the loss of an individual’s mental and physical resources, leading to energy exhaustion, anxiety, burnout, disappointment and other negative emotions, further reducing their job performance and leading to turnover and health problems (Lu and Tu, 2015). The empirical research confirms this view. For example, Adler and Koch (2017) and others found that employees undertake two kinds of countermeasures when work requirements exceed the work resources. One is coping with fatigue. Employees rely on their own subjective efforts to mobilize all the resources they can to maintain or meet work requirements. Such excessive efforts will cause fatigue. The second is a negative response in which employees are not willing to make full use of their resources to maintain or meet work requirements, and will actively reduce their awareness of work requirements, leading to performance degradation and other unprofessional behaviors. Therefore, when employees face higher work requirements and their available work resources are unable to meet this, there will be a negative impact on job performance. In the IT industry, the resources required by jobs often exceed the resources that employees can provide. The main reason is that the IT industry knowledge update speed is fast, and the staff’s learning intensity and work intensity are usually high, which may lead to fatigue coping and negative coping (Kun and Gadanecz, 2019). From this, we made the hypothesis H1, that the relationship between work engagement and job performance is an inverted U-shape.

### The Role of Psychological Capital in the Relationship Between Work Engagement and Job Performance

The JD-R model also proposes that work resources will buffer the physical and/or psychological consumption of work requirements, and regulate the relationship between work engagement and job performance. In the case of greater work resource support, job performance will increase accordingly. For example, Wang et al. (2012) found that social support and job development opportunities have a positive impact on job performance.

However, in the recent development of the information technology industry in terms of internal resources, the focus is on developing and utilizing the existing knowledge and experience of employees. That is, paying attention to the development of human capital and relatively ignoring the importance of psychological capital of programmers to the development of individuals and enterprises. Psychological capital, more so than human capital, can predict the job performance and positive work attitude and behavior of employees (Tian and Xie, 2010; Yin et al., 2018), and is more likely to be an adjustment variable on the relationship between work engagement and job performance. Therefore, when considering the JD-R theoretical model, many researchers have proposed taking psychological capital into account (Zhao et al., 2013). For example, Sun et al. (2014), when studying the JR-D theoretical model, considered psychological capital to be an internal resource for development that helps practitioners respond to various work requirements with a positive psychological state, and one that can effectively prevent and improve job burnout and finally, improve job performance. Psychological capital has increasingly been found to play a positive role in the relationship between work engagement and job performance (Qi and Wu, 2018). Psychological capital is usually regarded as an individual’s internal resources, which plays a positive role in individuals’ work efficiency (Luthans et al., 2008). In the IT industry, the positive role played by psychological capital is also being concerned by researchers (Sihag and Sarikwal, 2015). Therefore, we made the hypothesis H2, that psychological capital plays a moderate role in the relationship between work engagement and job performance.

## Materials and Methods

### Participants

Participants were programmers from 3 well-known IT companies in Nanjing. The reason for choosing them is that IT industry has a relatively fast updating knowledge, and programmers can best represent the working status of employees in “Internet plus” era. A total of 420 questionnaires were sent out and 399 valid questionnaires were collected. The response rate is 95%. Participants were aged 20–48 (*M* = 26.84, *S* = 5.82), of whom 271 were male and 128 were female. In total, 122 (30.6%) had worked for less than 1 year, 171 (42.9%) for 1–3 years and 106 (26.5%) for more than 3 years.

### Measures

#### Work Engagement

The Chinese version of the work engagement scale, developed by Schaufeli and Bakker (2004) and revised by Zhang and Gan (2005), was used to assess the level of employee work engagement from physical, emotional and cognitive perspectives. The scale consists of 15 items in total, for example, “I feel myself bursting with energy in my work,” “I am immersed in my work.” Each item was scored on a 6-point scale, ranging from 1 (never) to 6 (Always). The reliability of this scale is greater than 0.70, in this study, Cronbach’s α coefficient was 0.72. Confirmatory factor analysis showed that the fit indexes for χ^2^/df = 1.36, Tucker–Lewis index (TLI) = 0.99, comparative fit index (CFI) = 0.99, and root mean square error of approximation (RMSEA) = 0.030. The indicators of the model fit were accepted.

#### Job Performance

The measure of employee job performance was developed by Han et al. (2007) and it is suitable for the measurement of domestic knowledge workers’ job performance. There are 39 items, for example, “I complete my work in accordance with the requirements of the formal performance appraisal,” “I volunteer for duties that are not my own.” Each item was scored on a 5-point scale, ranging from 1 (Strongly disagree) to 5 (Strongly agree), the scale assess employee job performance across the four dimensions of innovation performance, relationship performance, learning performance and task performance. Part of the reason for choosing this scale is that enterprise programmers also belong to the category of knowledge employees. The other reason is that the four dimensions of this scale are closely matched to the work content of IT enterprise programmers. The Cronbach’s α coefficient of the job performance scale was 0.88, in this study, Cronbach’s α coefficient was 0.87. Confirmatory factor analysis showed that the fit indexes for χ^2^/df = 1.04, TLI = 0.99, CFI = 0.99, and RMSEA = 0.010. The indicators of the model fit were accepted.

#### Psychological Capital

The Chinese version of the psychological capital scale, developed by Luthans et al. (2008) and revised by Zhong et al. (2013). There are 24 items which are measured across the four dimensions of self-efficacy, hope, optimism and resilience on a six-point scale. for example, “I believe I can analyze long-term problems and find solutions,” “Currently, I am working energetically to accomplish my goals.” Each item was scored on a 6-point scale, ranging from 1 (Strongly disagree) to 6 (Strongly agree). The Cronbach’s α coefficient of Psychological capital scale was 0.89. In this study, the Cronbach’s α coefficient was 0.90. Confirmatory factor analysis showed that the fit indexes for χ^2^/df = 2.09, TLI = 0.90, CFI = 0.92, and RMSEA = 0.052. The indicators of the model fit were accepted.

#### Control Variables

We control some variables that can influence the research results, such as gender, working years and other demographic variables to maintain a balance. Environmental variables such as noise in the measurement process are excluded, and the experimenters and assistants are strictly trained to ensure that there is no error caused by human factors.

### Procedure

This study was approved by the ethical review boards of the authors’ institutions. Written informed consent was obtained from all participants before their enrollment in the study. They were informed that they could withdraw from the study at any time. Participants from three IT companies in Nanjing were gathered in a quiet place. After reading the instructions provided by the experimenter, they completed the questionnaire according to their recent job performance. After completing the task, they received ¥30 for taking part in the survey. Before the formal survey, we conducted a pilot test with about 100 IT employees, and found significant correlation among the three variables.

### Common Method Bias Control

The data of this study were collected by self-report, the could have been affected by common method bias, which might, in turn, decrease the validity of the results. So we used “process control” and “statistical control” for controlling for common method bias. Process control refers to control measures incorporated into the process of a study’s design and measurement by researchers (Yao and Yang, 2017). In this study, we kept strict principles of confidentiality and voluntarism, and asked participants to truthful answer each question. We used random sampling method to get participants and collect data in a closed environment, and recycled the questionnaires immediately after each survey was completed. These methods can effectively control the common method bias. In addition, statistical control involves “a statistical test that is applied after data collection” (Yao and Yang, 2017), and we used the Harman single factor test to test for common method bias. The results showed that eight factors had an eigenvalue greater than 1, and the first factor accounted for 25.42% of the variance, which is less than the critical standard of 40%. This shows that common method bias was not apparent.

## Results

### Descriptive Statistics

Before testing the hypothesis model, we conduct confirmatory factor analysis to evaluate the suitability of the research model, the result the fit indexes for χ^2^/df = 1.58, TLI = 0.91, CFI = 0.91, and RMSEA = 0.038. The indicators of the model fit were accepted.

The means, standard deviations, and correlation coefficients for each variable were calculated and presented in Table 1. Work engagement, job performance and psychological capital were all positively correlated.

**TABLE 1:** Means, standard deviations, and intercorrelations for the key study variables.

	*M* ± *S*	1	2	3
1 Work engagement	53.68 ± 12.32	1		
2 Job performance	100.90 ± 13.53	0.51***	1	
3 Psychological capital	79.15 ± 13.61	0.19***	0.62***	1

*N = 399, all tests were two-tailed.*

*p < 0.05, **p < 0.01, ***p < 0.001.

### The Test of the Relationship Between Work Engagement and Job Performance

**FIGURE 1:**
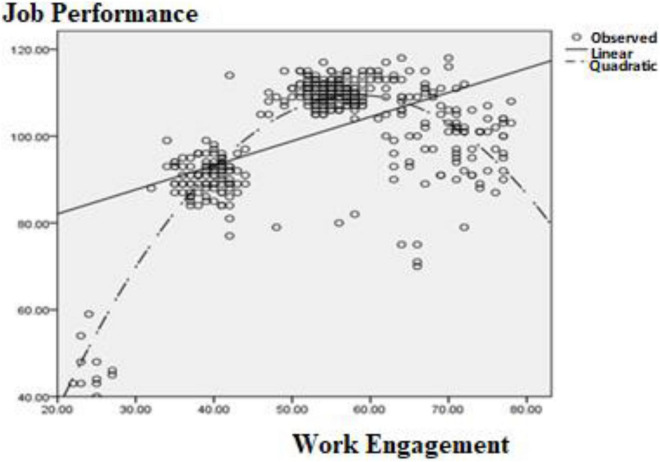
Plot models and the relationship between work engagement and job performance.

Regression analysis was used to assess the relationship between work engagement and job performance, and to compare the advantages and disadvantages of the linear and quadratic models. Results are shown in Table 2 where it can be seen that the linear and quadratic relationships between work engagement and job performance were both significant. In the linear model, work engagement could only explain 26% (*R*^2^ = 0.26) of the variation in job performance but in the quadratic model, work engagement could explain 72% (*R*^2^ = 0.72) of the variation of job performance, showing that the quadratic model was better than the linear model. The relationship between work engagement and job performance was an inverted U-shape (see Figure 1).

**TABLE 2:** Linear and curvilinear estimation of work engagement and job performance.

Model	Dependent	Independent	*R* ^2^	*F*	β	*t*
Liner	Job performance	Work engagement	0.26	138.78***	0.51	11.78***
Quadratic	Job performance	Work engagement	0.72	501.13***	−4.76	−25.30***

*p < 0.05, **p < 0.01, ***p < 0.001.

### The Moderating Effect of Psychological Capital

In this study, psychological capital, work engagement and job performance were all continuous variables, and the relationship between work engagement and job performance was an inverted U-shape. We adopted the regulatory analysis method of non-linear relationships described by Luo and Jiang (2014) relating to the questionnaire research method by regulating the high (one standard deviation higher than the average) and low (one standard deviation lower than the average) values for psychological capital, and the high (one standard deviation higher than the average), medium (average), and low (one standard deviation lower than the average) values for work engagement. The confidence intervals of the dependent variable (job performance) corresponding to the independent variable (work engagement) were calculated, respectively (*p* = 0.05). We used Mplus 6.0 to analyze the regulatory effect of the inverted U-shape relationship and the results are shown in Table 3. These show that when psychological capital is low, the 95% confidence interval for the job performance of participants with high, medium, and low work engagement almost overlapped. This shows that different levels of work engagement do not cause significant differences in job performance. However, when the psychological capital is high, the middle point of the confidence interval is higher than the other two points, showing an inverted U-shaped relationship. That is to say, only when psychological capital is high, do work engagement and job performance show a significant inverted U-shaped relationship, confirming a regulatory effect.

**TABLE 3:** The moderating effect of psychological capital.

Psychological capital	Work engagement	Job performance	Confidence interval (95%)
Low	Upper	74.62	[71.16, 78.31]
	Middle	75.30	[72.25, 80.43]
	Low	70.97	[68.23, 76.32]
Upper	Upper	83.21	[79.12, 86.21]
	Middle	108.53	[101.32, 116.51]
	Low	46.77	[45.19, 62.26]

**FIGURE 2:**
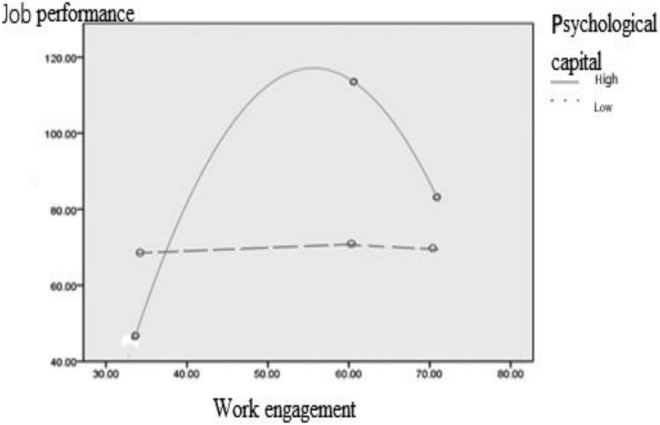
The moderating effect of psychological capital.

SPSS was used to draw the curve estimation model of the relationship between work engagement and job performance under the condition of high mental capital (the highest 27%) and low mental capital (the lowest 27%), and put the two models into the same coordinate axis. The results are shown in Figure 2, which more clearly shows the regulatory effect of mental capital on the relationship between work engagement and job performance, that is, for low mental capital. In the case of adjustment, the job performance of participants only slightly increased with the increase of work engagement and then decreased. In the case of high psychological capital adjustment, this took place before the work engagement reached the critical value. The job performance of IT enterprise programmers significantly increased with the increase of work engagement, and after exceeding the critical value of work engagement, the job performance decreased with the increase of work engagement.

## Discussion

In this study, programmers in the IT industry were selected as research participants to explore the relationship between work engagement and job performance, as well as the regulatory role of psychological capital. The results of the correlation analysis showed that there were significant positive correlations between work engagement, job performance, and psychological capital, and this indicated that there may also be positive relationships between work engagement, job performance and psychological capital. Through the analysis of the relationship between work engagement and job performance, we found that an inverted U-shaped relationship was more suitable for the data distribution than a linear relationship, meaning that the relationship between work engagement and job performance is not simply positive correlation. Appropriate work engagement is very important to job performance. This result is consistent with findings from some previous studies. For example, Demerouti et al. (2001) found that if the level of work engagement is too high, the relationship between work engagement and job performance will not be positive. Macey and Schneider (2010) also pointed out that to maintain long-term and stable job performance, employees cannot be in a high engagement state in a short period of time.

Through the analysis of the moderating role of psychological capital, we also found that there is a significant inverted U-shaped relationship between work engagement and job performance for individuals with high psychological capital. This shows that when individuals have a certain amount of psychological capital, higher job performance was associated with appropriate work engagement. However, for individuals with low psychological capital, job performance is always at a low level, and has a weak association with work engagement. The reasons for this may be twofold. First, psychological capital plays an important role in improving job performance, which will stimulate individuals to invest more efforts to participate in individual work (Qi and Wu, 2018). At the same time, psychological capital may produce more organizational citizenship behavior and promote performance. Second, when an individual has certain resources, particularly internal resources such as psychological capital, it can effectively buffer the adverse effects of work engagement on the individual, including anxiety, psychological exhaustion or burnout. We also found that under the same level of work engagement, individuals with higher psychological capital will have better job performance.

This research has made contributions in both theory and practice. In theory, it confirms the inverted “U” relationship between work engagement and job performance, and verifies the applicability of job requirement-resource model on Chinese cultural groups. At the same time, it makes a useful exploration on the theoretical model of IT employees’ job performance. The study also verified the moderating role played by psychological capital in the relationship between work engagement and work performance, which implies that individual work performance is not only related to the provision of good working conditions, but also closely related to the state of the individual. Psychological capital can be used as a resource to enhance performance.

In practice, job performance can be improved in three ways: first, by providing suitable working conditions to meet their needs so that they can devote more time and energy to their work; second, a reasonable match between people and jobs can improve performance. Thirdly, the psychological capital of employees is developed from within, thus improving the individual state.

The value of this article is the discovery that for individuals to achieve the highest job performance, a moderate level of work engagement is optimal, while individuals with higher psychological capital will have higher performance with the same work engagement. The disadvantage of this study is that performance is measured by self-report. Although it is more suitable for this study, it is different from the real situation of employee performance. In order to offset this limitation, we controlled the social desirability and possible memory bias of the participants, and asked them to evaluate their own situation in the last week and answer truthfully. Although it cannot completely eliminate the influence of social approval and memory bias, it can reduce the reaction bias to a certain extent. In the future, we can measure performance from the perspective of a third party to reduce errors. In addition, the results obtained from cross-sectional data are essentially a correlation, not a causal relationship. Therefore, this study only makes a possible inference on causality on the basis of correlation, and will use longitudinal data to reveal causality in the future.

The future research can be expanded on the following three aspects: first, explore the relationship between work engagement and job performance in the context of group, and consider the influence of group characteristics, such as collective psychological capital. Secondly, longitudinal research can be used to confirm the causal effect on the development of employee psychological capital and the improvement on employee performance. Finally, qualitative research can be used to explore the theoretical model of the impact process on how psychological capital can buffer the negative impact of excessive work engagement and how to improve job performance, and lay a foundation for future research in this field.

## Data Availability Statement

The original contributions presented in the study are included in the article/supplementary material, further inquiries can be directed to the corresponding author/s.

## Ethics Statement

This study was carried out following approval by the Ethics Committee of the Psychological Experiment Teaching Centre of Nanjing Normal University. All procedures performed in this study were in accordance with the ethical standards of authors’ institutional research committee and with the 1964 Helsinki Declaration and its later amendments or comparable ethical standards. Written informed consent was not required to participate in this study in accordance with the national legislation and the institutional requirements.

## Author Contributions

JY participated in the design, data collection, data analysis, data interpretation, and drafting the early version of the article. XQ and LY participated in the design and revising the article critically for better intrinsic logicality. XH participated in the design and drafting the early version of the article. YL participated in data analysis. All authors contributed to the article and approved the submitted version.

## Conflict of Interest

The authors declare that the research was conducted in the absence of any commercial or financial relationships that could be construed as a potential conflict of interest.

## Publisher’s Note

All claims expressed in this article are solely those of the authors and do not necessarily represent those of their affiliated organizations, or those of the publisher, the editors and the reviewers. Any product that may be evaluated in this article, or claim that may be made by its manufacturer, is not guaranteed or endorsed by the publisher.
